# Association of *ESX1* gene variants with non-obstructive azoospermia in Chinese males

**DOI:** 10.1038/s41598-021-84182-0

**Published:** 2021-02-25

**Authors:** Qian Ma, Ye Du, Xiaomin Luo, Jing Ye, Yaoting Gui

**Affiliations:** Guangdong and Shenzhen Key Laboratory of Male Reproductive Medicine and Genetics, Institute of Urology, Peking University Shenzhen Hospital, Shenzhen PKU-HKUST Medical Center, Shenzhen, People’s Republic of China

**Keywords:** Reproductive disorders, Urogenital diseases

## Abstract

Genetic factors are one of the most important causes of non-obstructive azoospermia (NOA). *ESX1* is an X-linked testis-biased expressed gene, and a potential biomarker for testicular sperm retrieval in NOA patients, yet few systematic studies have investigated its association with NOA. Here, we performed selected exonic sequencing in a large cohort of Chinese males, and four novel missense mutations (including one compound mutation), one novel synonymous mutation of ESX1 unique to NOA patients were identified. We analyzed the effects of *ESX1* mutations on cyclin A degradation and cell cycle progression by immunoprecipitation assay and flow cytometry, and found that the compound mutant p.[P365R; L366V] ESX1 compromised the stabilizing effect of ESX1 on polyubiquitinated cyclin A, thereby causing the failure of M phase arrest in cells. Further studies showed that the deleterious effect of the compound mutations on ESX1 protein function was attributed to p.P365R but not p.L366V alteration. The novel ESX1 mutation p.P365R might confer high risk for NOA in Han Chinese population, probably via affecting cell cycle control.

## Introduction

Infertility affects about 15% of couples and male factor accounts for nearly half of all infertility cases ^1^. Non-obstructive azoospermia (NOA) is the one of the most severe forms of male infertility, and is characterized by the absence of sperm in the ejaculates of NOA patients ^2^. The etiology of NOA includes genetic abnormalities, and infectious and environmental causes ^2–4^. A few studies have reported that genetic factors are involved in the development of NOA, including Y-chromosome microdeletions and rare mutations, based on familiar case reports and mouse model analysis ^5–9^. However, the molecular basis of NOA is still poorly understood.

Human *ESX1* is an X-linked paired-like homeobox gene, and is primarily expressed in the testis in adult human tissues ^10,11^. Early studies in mice showed that *Esx1* is essential for placental development ^12^. Following bioinformatic analysis of primate ESX1 suggested that rapid evolution has occurred especially on the C-terminal region ^13^. Specifically, the highly conserved homeodomain of mouse *Esx1* gene showed 65% identity with human *ESX1* ^11^. Besides, no significant sequence homology could be found outside the homeodomain region. In mouse, there are more than 30 X-linked homeobox genes (*Rhox* gene cluster) expressed in reproductive tissues and some are vital for spermatogenesis, such as *Rhox 5*. In contrast to this, on the human X chromosome, there are only two *RHOX* orthologs implicated in spermatogenesis. The X-linked male-specific genes usually evolve faster than genes located on autosomes, as they were confronted with more intense selective forces ^14,15^. It is possible that the human *ESX1* gene, especially the C-terminal region, undergone rapid evolution, has undertaken part of the murine *Rhox* functions in spermatogenesis since the split between hominids and rodents ^16,17^.

Studies based on clinical samples showed the expression level of ESX1 in testicular biopsies and seminal fluids was positively correlated with the presence of residual spermatogenesis in NOA men, indicating that ESX1 might be a potential predictor of successful sperm retrieval at surgery ^16,17^. It was also reported that in human cells and testis tissue, ESX1 protein is proteolytically cleaved into a N-terminal 45 kD fragment containing the homeodomain and a C-terminal 20 kD fragment containing a proline-rich region ^18^. The N-terminal fragment was localized in the nucleus and could transcriptionally repress the expression of human *K-ras* gene which in turn inhibits the growth of cancer cells harboring oncogenic *K-ras* ^11^. The C-terminal fragment was localized in the cytoplasm and could functionally inhibit the degradation of cyclins ^18^. Given the findings above, we speculated ESX1 might be involved in the regulation of cell cycle progression during human spermatogenesis.

In this study, we systematically investigated the association between *ESX1* variations and NOA. Selected exonic sequencing was performed to identify novel variations of *ESX1* in 766 NOA patients and 709 fertile controls. Bioinformatic analysis as well as functional studies were carried out to analyze the potential effects of these mutations on ESX1 function.

## Results

### Identification of *ESX1* mutations in patients with NOA

**Figure 1:**
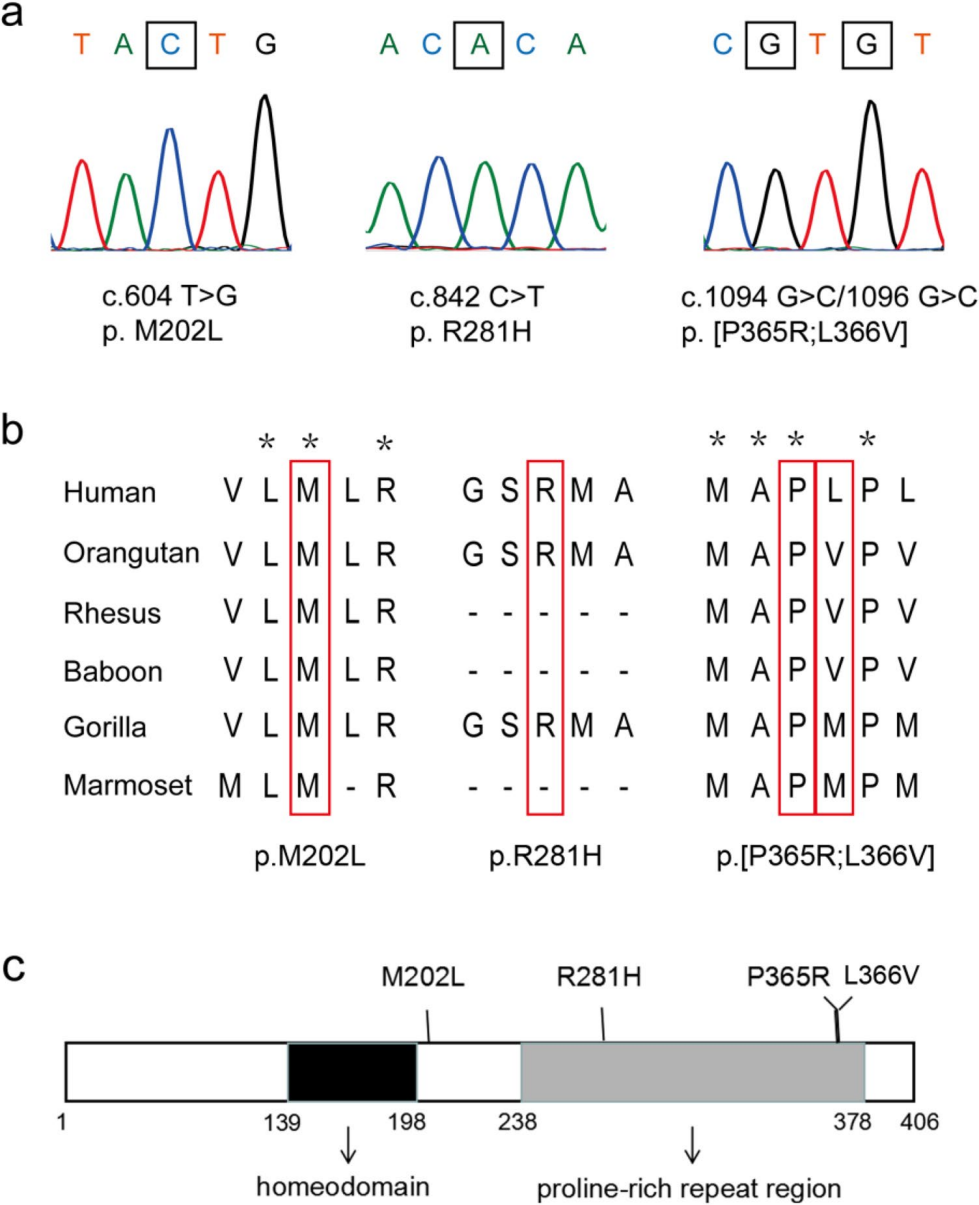
Missense mutations of *ESX1* identified in patients with NOA. (**a**) Four missense mutations were validated by PCR-Sanger sequencing. Mutation sites are marked with a black box. (**b**) The evolutionary conservation of each amino acid altered by these mutations was analyzed via multiple protein sequence alignments. (**c**) Schematic representation of ESX1 protein and its missense mutations.

In order to screen variations potentially associated with NOA, we employed massively parallel sequencing to identify exonic alterations of 654 infertility-related genes, including *ESX1* ^6–8^. As shown in Table 1, six missense mutations and two synonymous mutations were identified in *ESX1*. Of the six missense mutations, except for c.26A > C and c.480 A > C, four other missense mutations could only be detected in patients with NOA. Two adjacent missense mutations c.1094C > G and c.1096C > G were detected in the same patient W075. Clinical information of patients harboring these mutations was listed in Table 2. The patient carrying the p.[P365R; L366V] mutation had normal hormone levels, but an abnormal testis volume of 6 ml. The four missense mutations were further validated through PCR Sanger sequencing in corresponding patients with primers listed in Supplementary Table S1 (Fig. 1a). The evolutionary conservation of the amino acids altered by the missense mutations mentioned above were further analyzed by multiple sequence alignments of ESX1 with its orthologs in different species. The results showed that amino acids at 202 and 365 were highly evolutionarily conserved while amino acids at 281 and 366 were not (Fig. 1b). As is shown in Fig. 1c, except for p.M202L, the three other missense mutations unique to NOA patients were all located in the proline-rich repeat motif of the C-terminal region. Based on bioinformatic assessment of these variants, the mutation p.P365R was predicted to be damaging by multiple in silico predictors including SIFT ^19^, PolyPhen-2 ^20^, MutationAssessor and Combined Annotation Dependent Depletion (CADD) (Supplementary Table S2). As p.P365R and p.L366V were found in the same one patient, we firstly investigated these two mutations as compound mutations in the following functional studies.

**Table 1:** Mutations of *ESX1* identified in fertile men and patients with NOA.

No	Genomic location		Nucleotide variants		Amino acid changes	Fertile men (n = 709)	Patients (n = 776)	Allele Frequency in dbSNP
**Missense mutations**								
1	ChrX:103,499,505		c.26 A > C		p.H9P	1	0	0.000008 (rs1195713986)
2	ChrX:103,498,861		c.480 A > C		p.E160D	1	0	not found
3	ChrX:103,495,526		c.604 A > C		p.M202L	0	1	0.000006 (rs1556394172)
4	ChrX:103,495,288		c.842 G > A		p.R281H	0	1	0.00003 (rs782771126)
5	ChrX:103,495,036		c.1094 C > G		p.P365R	0	1	0.00006 (rs782108131)
6	ChrX:103,495,034		c.1096 C > G		p.L366V	0	1	0.0011 (rs782593165)
**Synonymous mutations**								
7	ChrX:103,498,888		c.453 G > A		None	1	0	0.00007 (rs782337186)
8	ChrX:103,494,921		c.1209 T > C		None	0	1	0.00012 (rs375756428)

**Table 2:** Clinical information of NOA patients with missense mutations in *ESX1*.

Nucleotide changes	Sample IDs	Age	Testicular volume (ml)	FSH (mIU/ml) [1.5–12.5]	LH (mIU/ml) [1.7–8.6]	T (ng/ml) [2.5–8.0]	Status of spermatogenesis
c.604 A > C	W568	28	10	26.70	10.40	NA	Arrested at spermatocytes
c.842 G > A	W245	31	5	14.10	3.23	2.30	SCO
c.1094 C > G; c.1096 C > G	W075	26	6	10.78	5.30	3.41	SCO

*FSH*, follicle stimulating hormone; *LH*, luteinizing hormone; *T*, testosterone; *SCO*, Sertoli cell only *NA*: not available.

### p.[P365R; L366V] ESX1 failed to inhibit degradation of cyclin A

**Figure 2:**
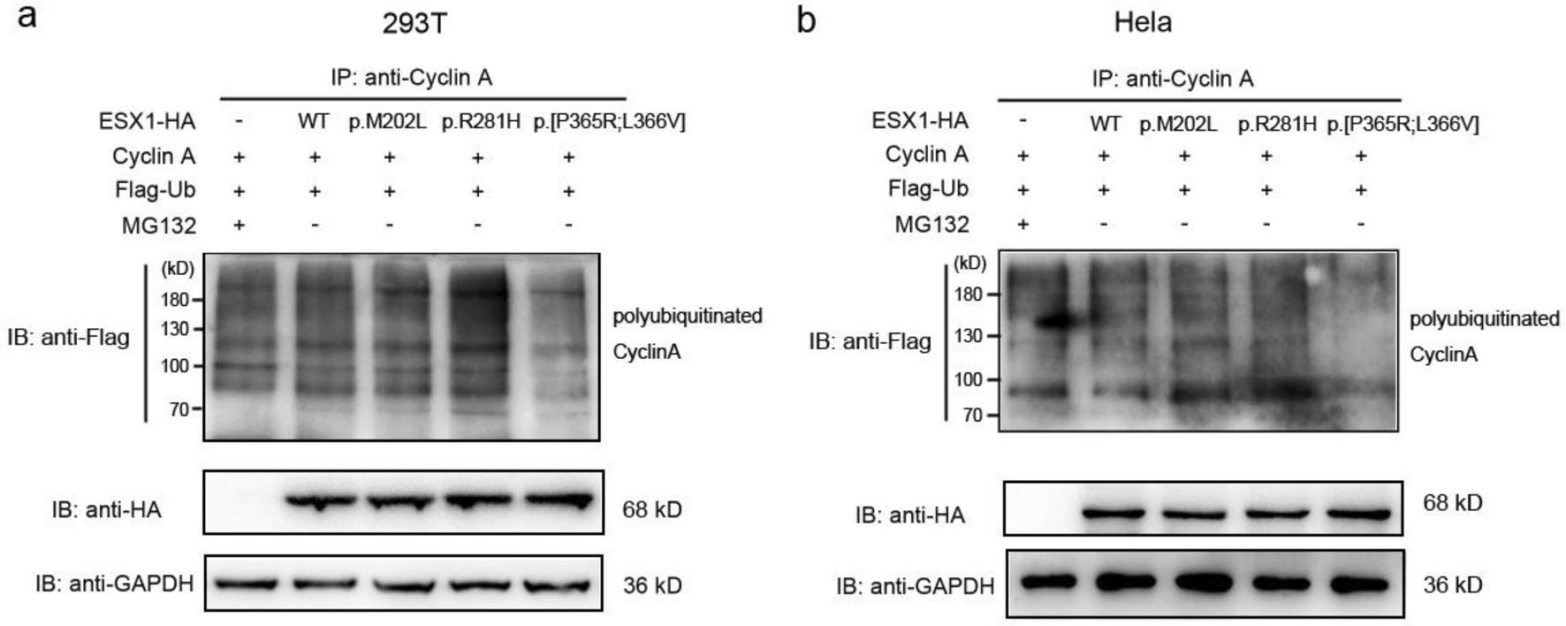
The p.[P365R; L366V] mutation compromised the stabilizing effect of ESX1 on polyubiquitinated cyclin A. Cyclin A and Flag-Ubiquitin were transfected into 293 T (**a**) or Hela (**b**) cells together with wild type or mutant ESX1-HA expression vectors, as indicated. Cells transfected with empty vector of pcDNA 3.1-HA and further treated with MG132 were used as positive control. Cell lysates were then prepared and immunoprecipitated with anti-Cyclin A antibodies. Cyclin A polyubiquitination was detected with anti-Flag antibody. The grouping of blots cropped from different parts of the same gel, or from different gels, fields, or exposures were divided by black lines. Compared with wild type ESX1, polyubiquitination of cyclin A was significantly decreased by the p.[P365R; L366V] mutation.

Cyclins are degraded through the ubiquitin-dependent proteasome pathway ^21^. It was reported that the cleavable C-terminal region of human ESX1 could inhibit cyclin degradation through stabilization of polyubiqutinated forms of cyclins ^18^. Then, to test whether these identified mutations affect cyclin accumulation, cyclin A and Flag-ubiquitin expression vectors were coexpressed with wild type (WT) or mutant ESX1-HA in 293 T (Fig. 2a) and Hela (Fig. 2b) cells. ESX1 protein could be detected by an anti-HA antibody efficiently when fused with an HA tag originating from hemagglutinin. The polyubiquitinated cyclin A was detected with an anti-Flag antibody after the cell lysates were immunoprecipitated by anti-cyclin A antibody. Cells transfected without ESX1-HA but treated with proteasome inhibitor MG132 were used as positive control. As shown in Fig. 2, like wild type ESX1-HA and positive control, a polyubiquitinated ladder of cyclin A could be detected in cells over-expressed with p.M202L ESX1 and p.R281H ESX1. But the polyubiquitinated cyclin A was significantly diminished when equivalent p.[P365R; L366V] ESX1 was expressed (Fig. 2a,b, last lane). The images of full-length blots were included in Supplementary Figs. S1 and S2. These results suggested that the p.[P365R; L366V] mutation altered the inhibitory effect of wild type ESX1 on cyclin A degradation.

**Figure 3:**
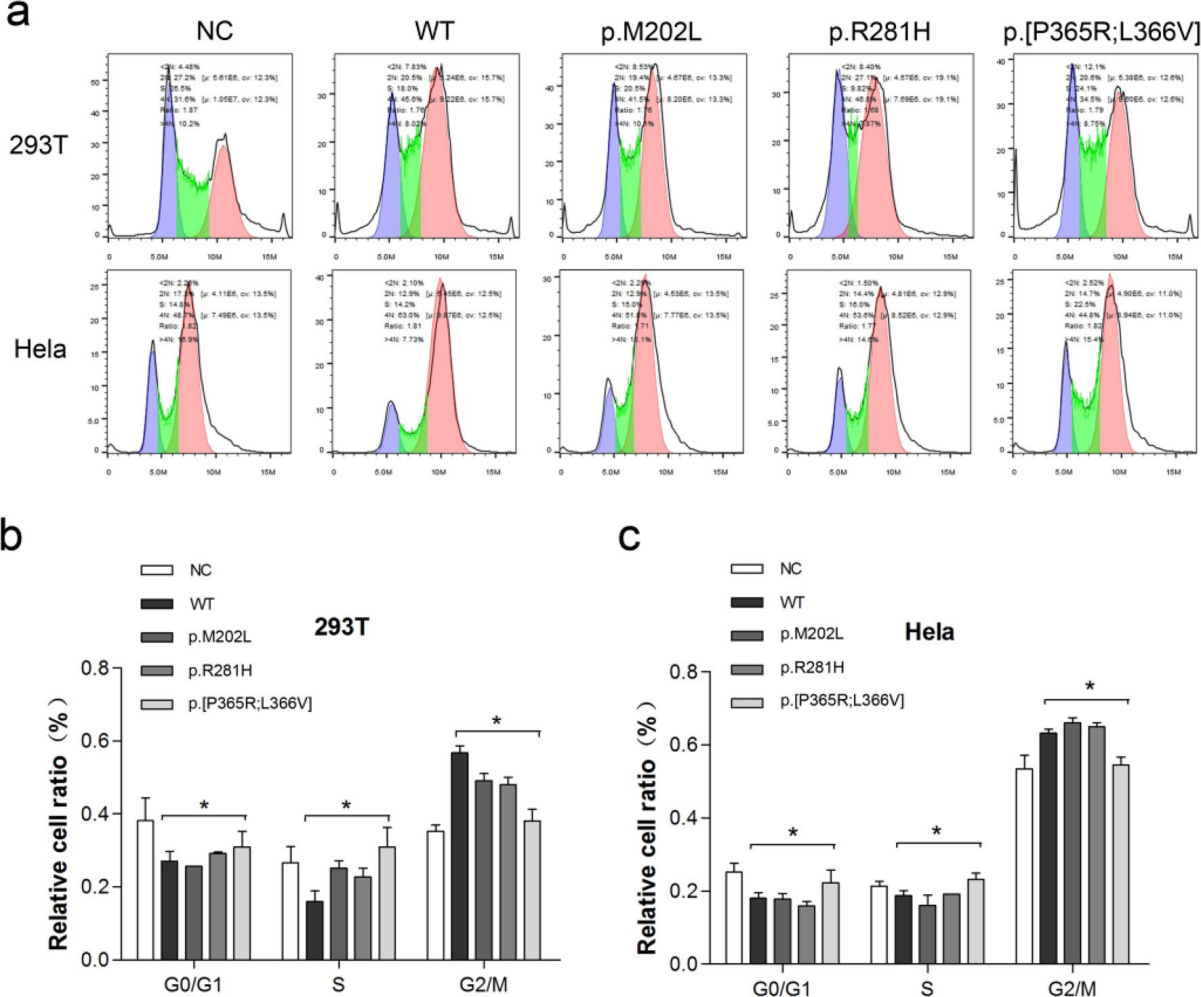
p.[P365R; L366V] ESX1 disrupted the inhibitory effect of ESX1 on cell cycle of 293 T and Hela cells. Wild type or mutant ESX1-HA expression vectors were transfected into cells as indicated. Empty vector was used as negative control (NC). Cells were synchronized at S phase with thymidine blocking for 36–48 h, then washed with PBS and treated with cytosine for another 6 h. (**a**) Cells were harvested and cell cycle profiles were analyzed by flow cytometry and the FlowJo software. The G0/G1, S, or G2 phase was marked with blue, green and pink colour, respectively. Relative cell ratio of different phases in 293 T (**b**) and Hela (**c**) generated by FlowJo from at least three independent experiments were analyzed statistically using GraphPad Prism 5 software. Compared with wild type ESX1, p.[P365R; L366V] ESX1 lost the ability to arrest cells at M phase. * *P* < 0.05.

**Figure 4:**
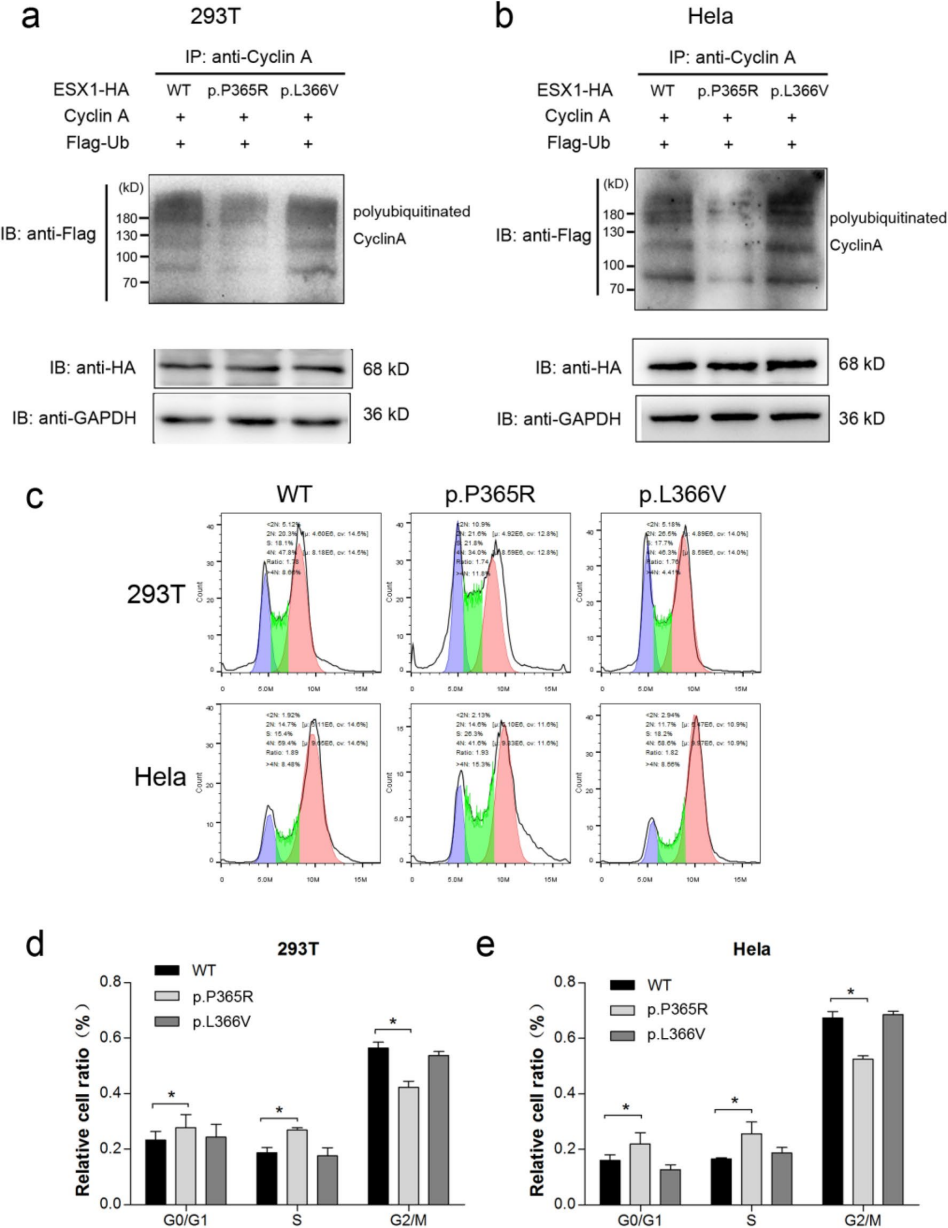
The p.P365R but not p.L366V mutation attributes to the deleterious effect on ESX1 function. p.P365R or p.L366V ESX1 overexpression plasmids were transfected into 293 T and Hela cells as indicated. Wild type ESX1 was used as control. After synchronization as described above, cells were either lysed for IP to detect the polyubiquitination of cyclin A (**a** and **b**), or subjected to flow cytometry (**c**). Relative cell ratio of different phases in 293 T (**d**) and Hela (**e**) were analyzed statistically using GraphPad Prism 5 software. The grouping of blots cropped from different parts of the same gel, or from different gels, fields, or exposures were divided by black lines. Compared with wild type ESX1, p.P365R but not p.L366V failed to stabilize polyubiquitinated cyclin A, and further lost the ability to arrest cells at M phase. * *P* < 0.05.

**Figure 5:**
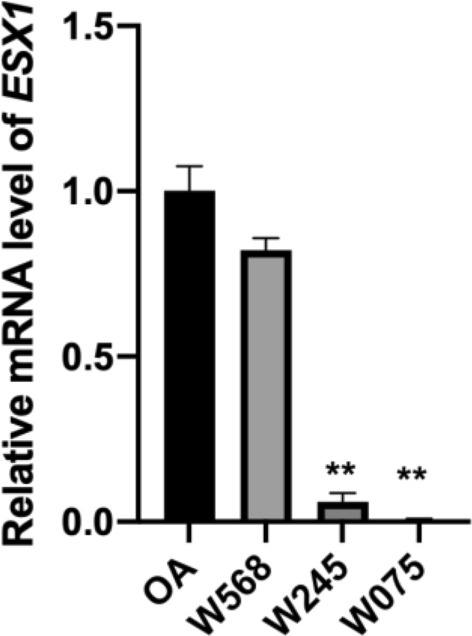
*ESX1* mRNA levels in NOA patients harboring ESX1 mutations. RNA was extracted from FFPE testicular samples and then reverse-transcribed into cDNA. Compared with patient with OA, ESX1 mRNA level was substantially decreased in patients harboring the p.R281H (W245) or p.[P365R; L366V] (W075) mutation. ** *P* < 0.01.

**Figure 6:**
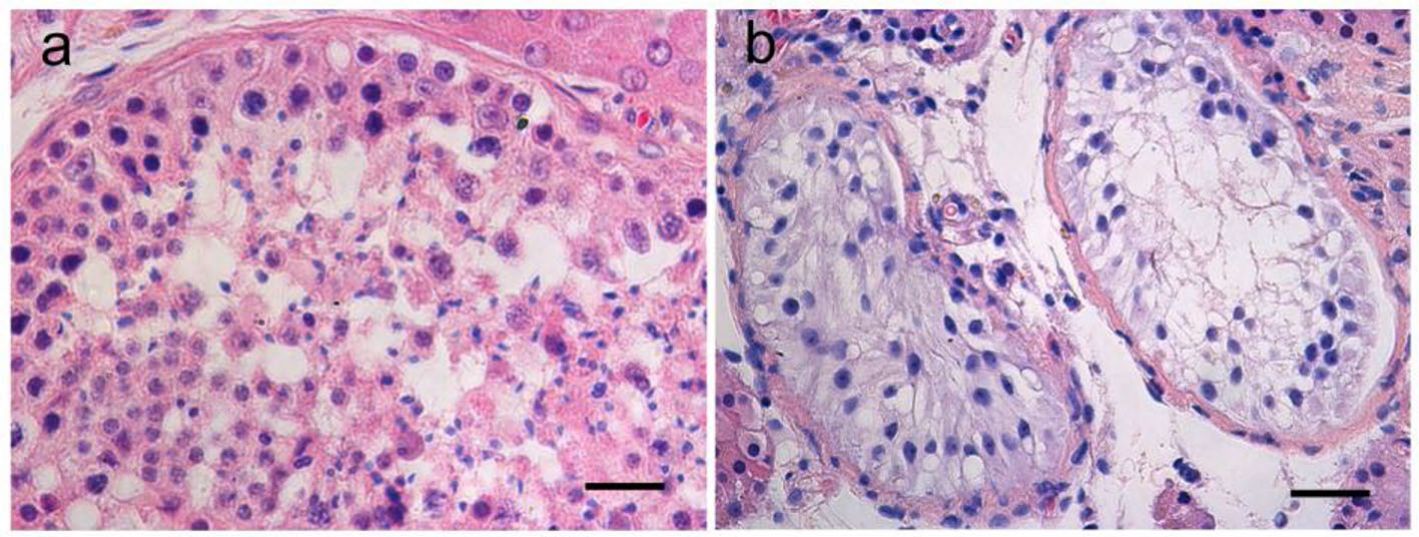
Hematoxylin and eosin staining of testis sections from (**a**) a patient with obstructive azoospermia (OA), and (**b**) the NOA patient carrying the ESX1 p.[P365R; L366V] mutation. Only Sertoli cells could be detected within the testis tissue of this NOA patient (Sertoli-cell-only-syndrome, SCO), whereas the tissue of the OA patient displayed full spermatogenesis. Scale bar: 50 μm.

### p.[P365R; L366V] ESX1 impaired the inhibitory effect of ESX1 on cell cycle

Cyclins are key regulators of cell cycle progression and cyclin accumulation could cause cell cycle arrest ^22^. Overexpression of ESX1 was reported to prevent degradation of polyubiquitinated cyclins in human cells and thereby provoke M phase arrest ^18^. To further explore the effect of the mutated ESX1 on cell cycle regulation, we performed flow cytometry to detect the ratio of cells in different phases during a cell cycle. The original FCS data was analyzed using the FlowJo analysis program, and the representative output histograms showing phase distribution were presented in Fig. 3a. FlowJo has a Cell Cycle Platform that uses multiple models to fit the data, constrains the fitting parameters, and automatically calculates the percentage of cells in the G1, S, or G2 phases. As shown in Fig. 3a, compared with cells transfected with empty vector (negative control, NC), transient expression of WT ESX1 in 293 T and Hela cells synchronized caused a dramatic increase in G2/M phase cells. The mutations of p.M202L and p.R281H also manifested the same inhibitory effect on the cell cycle. However, compared with WT, the ratio of G2/M phase cells transfected with p.[P365R; L366V] ESX1 was significantly decreased with P value < 0.05 as shown in Fig. 3b,c. These results showed that p.[P365R; L366V] ESX1 failed to arrest cells at the M phase.

### p.P365R but not p.L366V was deleterious to ESX1 function

In order to figure out if the deleterious effects of the compound p.[P365R; L366V] mutation was attributed to either of those two variants alone, functional studies on the two variants were performed separately. As shown in Fig. 4a,b, compared with WT ESX1, the polyubiquitinated cyclin A ladder was significantly weaker in cells overexpressing p.P365R ESX1, which means the stabilizing effect of ESX1 on cyclin A was diminished by p.P365R mutation (Supplementary Fig. S3). The effect of p.P365R ESX1 on cell cycle arrest was also changed as revealed by flow cytometry (Fig. 4c–e), whereas no significant change on ESX1 function was detected in cells overexpressing p.L366V. Similar effects of the compound mutant and the p.P365R variant alone, showed that p.P365R but not p.L366V contributed to the deleterious effect on ESX1 function.

### Impact of *ESX1* variants on gene expression

In order to evaluate the effect of these variations on *ESX1* expression, RNA was extracted from the formalin fixed paraffin embedded (FFPE) testicular samples. Sample from obstructive azoospermia (OA) was used as control. The results of RT-qPCR showed that the mRNA level of *ESX1* was substantially decreased in patient W245 harboring the p.R281H variation or patient W075 with the p.[P365R; L366V] mutation (Fig. 5).

### Testicular biopsy analysis

Compared with the patient with obstructive azoospermia, the testicular biopsy of the patient carrying the p.[P365R; L366V] mutation confirmed the diagnosis of NOA. Only Sertoli cells were observed in the seminiferous tubules (SCO) and no germ cells could be found, which is consistent with the quite low testis volume of the patient (Fig. 6). The arrested maturation at the level of spermatocytes or SCO were also observed in patient W568 harboring the p.M202L mutation or patient W245 with the p.R281H mutation, respectively (Supplementary Fig. S4).

## Discussion

Previous studies showed that *ESX1* was specifically expressed in the testis tissue in adult human. The mRNA expression level of *ESX1* in testicular biopsies and seminal fluids was positively correlated with the residual spermatogenesis of NOA patients ^16,17^. Yet whether genetic defects of *ESX1* were associated with NOA is still unknown. In this study, we found although rare, *ESX1* gene variations unique to Han Chinese patients with NOA did exist via massively parallel sequencing in a large cohort of subjects, and no mature sperm could be found in the testicular biopsy of those patients. Among the four missense mutations specifically identified in NOA patients, the p.P365R mutation located in the proline-rich region of the C-terminal fragment, might confer a high risk for NOA in Chinese males.

Bioinformatic analysis was employed via multiple in silico predictors to evaluate the potential pathogenicity of these variations. Due to the differences based on the predicting principle, the outcome is not always concordant. For example, SIFT is an algorithm using sequence homology to predict whether a substitution affects protein function ^19^. PolyPhen-2 uses eight sequenced-based and three structure-based predictive features which were automatically selected by an iterative greedy algorithm ^20^. Of those six predictors used, four gave supportive results that the p.P365R mutation is a functional alteration. When the p.P365R mutation occurred, the original highly conserved proline is replaced by an alkaline residue, which might be deleterious to the configuration of ESX1 protein.

Cyclins are key regulators of cell cycle control and play an essential role in both mitosis and meiosis during germ cell production. Mice lacking cyclin A1 and cyclin A2 exhibited male sterility and early embryonic lethality, respectively ^23,24^. Our IP results showed that cyclin A could be successfully immunoprecipitated when ESX1 mutants overexpressed, suggesting that these mutations have no significant influence on the interaction between ESX1 and cyclin A. However, the polyubiquitination of cyclin A was decreased when p.P365R ESX1 was overexpressed, which means the ability to prevent the degradation of polyubiquitinated cyclin A by the proteosome pathway was affected ^18^. Cyclin A is transcriptionally activated in late G1 phase, then starts to accumulate in S phase. The consequence of compromised stability is that cyclin A might not stay that long in G2/M phase. According to the Human Protein Atlas, ESX1 is mainly expressed in the spermatogonia in human testis tissues (https://www.proteinatlas.org/ENSG00000123576-ESX1/tissue/testis). Cyclins also play an important role in the proliferation of spermatogonia, therefore, the p.P365R mutation might exert a deleterious effect on spermatogonia proliferation during spermatogenesis, which further leads to spermatogenesis defects. This is consistent with the low testis volume and the SCO phenotype of the NOA patient harboring the p.P365R mutation.

According to the single cell expression profile revealed by the Human Protein Atlas, *ESX1* is specifically expressed in the spermatogonia of adult human testis. For patients W245 and W075, only Sertoli cells were observed in the testis. Therefore, their *ESX1* mRNA levels were quite low.

The reproductive endocrine levels are regulated by a complex interplay between positive feedback and negative feedback from hormones within the hypothalamic-pituitary–gonadal axis, and the resulting serum FSH levels are expected to reflect both the pituitary and testicular function in physiological and pathological conditions ^25^. Decreased FSH levels are found in sterile patients with pituitary dysfunction whose T level is also low. Elevated FSH levels are usually found in NOA patients, as is shown in patients W568 and W245. However, to what extent the FSH level is related to testis histology still remains a question ^25^. Histological examination of a testicular biopsy from the patient W075 revealed Sertoli-cell-only-syndrome (SCO). Yet his FSH level (10.78 mIU/ml) is on the upper border of the normal range, which might be quite high to the patient W075 himself.

Different from other diseases, the outcome of NOA is infertility, which means it is almost impossible to perform large scale pedigree-based study. In most cases, male infertility-causing mutations are de novo or from maternal inheritance. By employing a feasible strategy which based on a large number of sporadic cases and genomic sequencing technology, we have identified series of novel pathogenic mutations in several genes in 776 NOA patients. *ESX1* is just one of them. Through analyzing the carrying rate of these mutations, we noticed that most mutations rarely occurred even within the NOA population ^6,7,26,27^. Single-locus based logistic regression analysis cannot identify any associated variant with statistical significance, but the gene-based and pathway-based analysis indicated that excess of case-unique variants was significantly enriched in several groups of genes and pathways ^6^. This is mainly because these mutations which lead to serious infertility failed to pass on to next generation. In the present study, we identified the novel p.P365R mutation in ESX1, of which the minor allele frequency is 0.00003579 in gnomAD database (https://gnomad.broadinstitute.org/). Closer inspection indicated that p.P365R was only identified in four European females as heterozygous variant carriers, but never found in male or other populations. The familial segregation analysis of *ESX1* variants is of good value in confirming the relationship between ESX1 mutation and azoospermia, yet we lost touch with the patients who were enrolled in this study more than ten years ago. Besides, these functional studies were performed in vitro which may not sufficiently reflect the functions of ESX1 in vivo. Thus, more verification experiments including genetic screening in larger NOA population and functional study in model animal will be further performed in the follow-up studies.

Spermatogenesis is a complex process which requires stringent control of cell cycle to ensure self-renewal and differentiation of various germ line cells. In this study, we have identified the novel missense mutation p.P365R in ESX1 potentially associated with NOA in a large cohort of Chinese patients through selected exonic sequencing combined with functional studies systematically. Nevertheless, more studies are still needed to thoroughly investigate the role of ESX1 in male infertility.

## Methods

### Subjects

In total, 1880 Han Chinese patients with azoospermia were recruited for this study from the Center of Reproductive Medicine, Tongji Medical College, Huazhong University of Science and Technology, from January 2007 to October 2011. Among them, 776 patients meeting the following criteria for NOA diagnosis were selected for further study: (1) no sperm detected in the pellets of semen samples on 3 different occasions; (2) no obstruction, inflammation, or injury of the reproductive system or pelvic cavity; and (3) no karyotypic abnormality or Y-chromosome microdeletion ^6,28,29^. 709 fertile Han Chinese men who had fathered at least one child without assisted reproductive techniques, were recruited as controls from the Center of Physical Examination, Peking University Shenzhen Hospital. After a panel resequencing study and quality control steps, 776 patients aged 24 to 46 years (average of 30.6 years) and 709 fertile men aged 29 to 51 years (average of 35.6 years) were available for further analysis.

### Ethics declarations

This study was approved by the ethics committee of Peking University Shenzhen Hospital and Tongji Medical College in accordance with the Declaration of Helsinki (Approval number: 20090018). Informed, written consents were obtained from all participants.

### Sequencing and mutational analysis of *ESX1* gene

The selected exonic sequencing and data analysis were performed in Beijing Genomics Institute at Shenzhen as described previously. Briefly, genomic DNA from peripheral blood samples was extracted using the QIAamp DNA Blood Midi Kit (QIAGEN, 51,185). The exon capture was performed using the NimbleGen custom array ^6–8^. Sequencing (paired-end 90-base pair reads) was performed on an Illumina Hiseq 2000 platform using recommended protocols from the manufacturer. After removing the low-quality bases and adaptor sequences, the sequencing reads were aligned against the human reference genome (NCBI build 37.1, hg19) using the SOAPaligner software (2.21).

Variations within *ESX1* were further validated by polymerase chain reaction (PCR) and Sanger sequencing. Primers F1 and R1 were used to detect ChrX:103495526 T > G; primers F2 and R2 were used to detect ChrX:103495288 C > T, ChrX:103495036 G > C and ChrX:103495034 G > C. Detailed primer sequences were listed in Supplementary Table S1.

### Plasmid construction and cell transfection

Human full-length wild type *ESX1* cDNA or its mutants synthesized were subcloned into pcDNA3.1-HA vector respectively, so that ESX1 protein overexpressed could be detected by an anti-HA antibody. The Cyclin A expression plasmid was obtained from GeneCopoeia. Human ubiquitin cDNA was synthesized and inserted into pcDNA3.1-Flag.

The 293 T and Hela cells were cultured in Dulbecco modified Eagle medium supplemented with 10% fetal bovine serum. All transfections were performed with Lipofectamine 3000 (Invitrogen, L3000015) according to the manufacturer’s guidelines.

### Immunoprecipitation (IP)

For immunoprecipitation, 2 μg wild type or mutant ESX1 plasmid, 0.2 μg cyclin A, and 0.2 μg Flag-ubiquitin expression vectors were cotransfected into HEK293T and Hela cells ^18^. Twelve hours after transfection, S phase synchronization was performed by treating cells with 2 mM thymidine for 36 h ^30^. Then, cells were washed with PBS for three times, incubated with medium, containing cytosine, for another 6 h. Cells transfected with cyclin A and Flag-ubiquitin were treated with 10 μg/ml MG132 as positive control. Immunoprecipitation was performed with Co-IP kit (Pierce, 88,805) under manufacturer’s instructions. Briefly, cells were lysed in IP lysis buffer and centrifugated at 12,000 rpm for 30 min. Supernatants were incubated under gentle rotation with magnetic Dynabeads, prebound to an anti-cyclin A antibody at 4 °C overnight. Beads were washed three times with lysis buffer and eluted with elution buffer. The immunoprecipitates were boiled with SDS loading buffer and subjected to the SDS-PAGE. The polyubiquitinated cyclin A was detected by an anti-Flag antibody. Resource for all antibodies used were listed in Supplementary Table S3.

### Western blot

Cell lysates or immunoprecipitates were subjected to the SDS-PAGE, and transferred to polyvinylidene fluoride membranes. The membranes were blocked with 5% nonfat milk in TBST buffer (20 mmol/L Tris–HCl, pH 7.5, 150 mmol/L NaCl, 0.1% Tween 20) and incubated with the anti-Flag antibody (1:1000), the anti-HA antibody (1:1000) or the anti-GAPDH antibody (1:2000) at 4 °C overnight. Then, membranes were washed 3 times with TBST buffer and incubated with horseradish peroxidase-labeled secondary antibody at room temperature for 1 h. Protein bands were visualized by chemiluminescence using Enhanced chemiluminescence (ECL) reagents (Millipore, WBKLS0500).

### RNA extraction and RT-qPCR

RNA from three FFPE testicular samples of NOA patients with ESX1 variants and one OA patient was extracted using an RNeasy FFPE Kit (QIAGEN, 73,504). cDNA was synthesized using the PrimeScript RT Master kit (Takara, RR037A) with random primers and oligo dT primers. RT-qPCR was carried out using the SYBR Premix EX Taq II PCR Kit (Takara, RR820A) following the manufacturer’s instructions on the Roche Lightcycler 480 Real-Time PCR System. Data were calculated according to the Applied Biosystems comparative Ct method. The primers used for human ESX1 were as follows: 5′-AACTTACCGTGACCTCGCTG-3′ (forward primer) and 5′-TCCGTGCCAACGTTGTTTTC-3′ (reverse primer); the primers used for human GAPDH were as follows: 5′-GAATGGGCAGCCGTTAGGAA-3′ (forward primer) and 5′-AAAAGCATCACCCGGAGGAG-3′ (reverse primer).

### Flow cytometry

293 T and Hela cells were transfected with 2 μg wild type ESX1 or equivalent ESX1 variants using Lipofectamine 3000 ^18^. Twelve hours later, cell synchronization and releasing were performed as aforementioned. Then, cells were harvested and fixed with 70% ethanol overnight at  − 20 °C. Samples were analyzed by flow cytometry (BD Accuri C6 Plus, BD Company) after staining with PI, complexed with RNase solution (Invitrogen, F10797). The data were analyzed using the FlowJo V10 analysis program (Treestar). FlowJo has a Cell Cycle Platform that uses multiple models to fit the data, constrains the fitting parameters, and automatically calculates the percentage of cells in the G1, S, or G2 phases. Briefly, the FCS data was dragged onto the interface. Then, cells along the most dense diagonal were gated to exclude potential debris or agglomerates via two serial gatings. Finally, the subpopulation was analyzed by selecting the Cell Cycle task. The ratios of phase distribution automatically generated by FlowJo from at least three independent experiments were analyzed statistically via GraphPad 5.

### Histopathology

Testis tissues collected from patients with azoospermia were fixed in 4% paraformaldehyde solution and sectioned into slides in the pathology department. Slides were stained with hematoxylin and eosin and then examined using light microscopy.

### Statistical analysis

Data are shown as the mean ± standard error of the mean (SEM) of values obtained in at least 3 independent experiments. The statistical significance of the differences between two groups was determined by Student’s *t* test using GraphPad Prism 5 statistical software with statistical significance adopted at 5%.

## Supplementary information

Supplementary information.

## Data Availability

All data included in this study are available upon request by contact with the corresponding author.
