# Supplementary material for: Association of ESX1 gene variants with non-obstructive azoospermia in Chinese males
Source: Sci Rep. 2021 Feb 25;11:4587. doi: 10.1038/s41598-021-84182-0 (PMC7907365; doi:10.1038/s41598-021-84182-0)
Supplement: Supplementary file 1 — Supplementary information. [file 41598_2021_84182_MOESM1_ESM.pdf]

Supplementary information

Association of *ESX1* gene variants with non-obstructive azoospermia in Chinese males

Qian Ma[1], Ye Du[1], Xiaomin Luo[1], Jing Ye[1]*, Yaoting Gui[1]*

[1]Guangdong and Shenzhen Key Laboratory of Male Reproductive Medicine and Genetics, Institute of Urology, Peking University Shenzhen Hospital, Shenzhen PKU-HKUST Medical Center, Shenzhen, P.R. China

*** Correspondence:** Yaoting Gui, E-mail: guiyaoting2007@aliyun.com;

Jing Ye, E-mail: ye2013j@163.com.

Table S1 Primers used to validate *ESX1* missense mutations in corresponding NOA patients.

Primers	Sequence (5'-3')
F1	CCTGTTGCAGTTTAAAGTCTCAGTG
R1	TAGGTAGTTGTGGCACCAGATGAAC
F2	CACCTGTTGCAGTTTAAAGTCTCAG
R2	TAACTGTTGATGACAGGGGCC

Table S2 Effects of ESX1 missense mutations on the protein function predicted by multiple in silico predictors.

No.	Nucleotide changes	Amino acid changes	PolyPhen-2	SIFT	I-Mutant	Mutationtaster	Mutation Assessor	CADD
1	c.26 A > C	p.H9P	Possibly damaging (0.666)	Damaging	0.12	polymorphism	Low (0.975)	9.515
2	c.480 A > C	p.E160D	Possibly damaging (0.911)	Damaging	-0.73	polymorphism	Neutral (0.685)	15.88
3	c.604 A > C	p.M202L	Benign (0.296)	Damaging	-1.08	polymorphism	Medium (2.16)	8.075
4	c.842 G > A	p.R281H	Probably damaging (0.978)	Tolerated	-0.71	polymorphism	Low (1.1)	0.009
5	c.1094 C > G	p.P365R	Probably damaging (0.985)	Damaging	-0.23	polymorphism	Medium (2.095)	15.56
6	c.1096 C > G	p.L366V	Benign (0.000)	Tolerated	-0.53	polymorphism	Neutral (-1.645)	0.002

I-Mutant: DDG<-0.5: Large Decrease of Stability; DDG>0.5: Large Increase of Stability; -0.5⩽ DDG⩽0.5: Neutral Stability.

MutationAssessor: predicted functional (high, medium), predicted non-functional (low, neutral).

Table S3 Resource for all antibodies used.

Antibodies	Source	Identifier
Rabbit monoclonal anti-HA	Cell Signaling Technology	Cat#3724
Mouse monoclonal anti-cyclin A2	Abcam	Cat#ab38
Mouse monoclonal anti-Flag	Sigma	Cat#F1804
Rabbit monoclonal anti-GAPDH	Abcam	Cat#16891

Figure S1. Full-length images of Western blots detecting the effect of ESX1 mutations on cyclin A polyubiquitination (Figure 2).

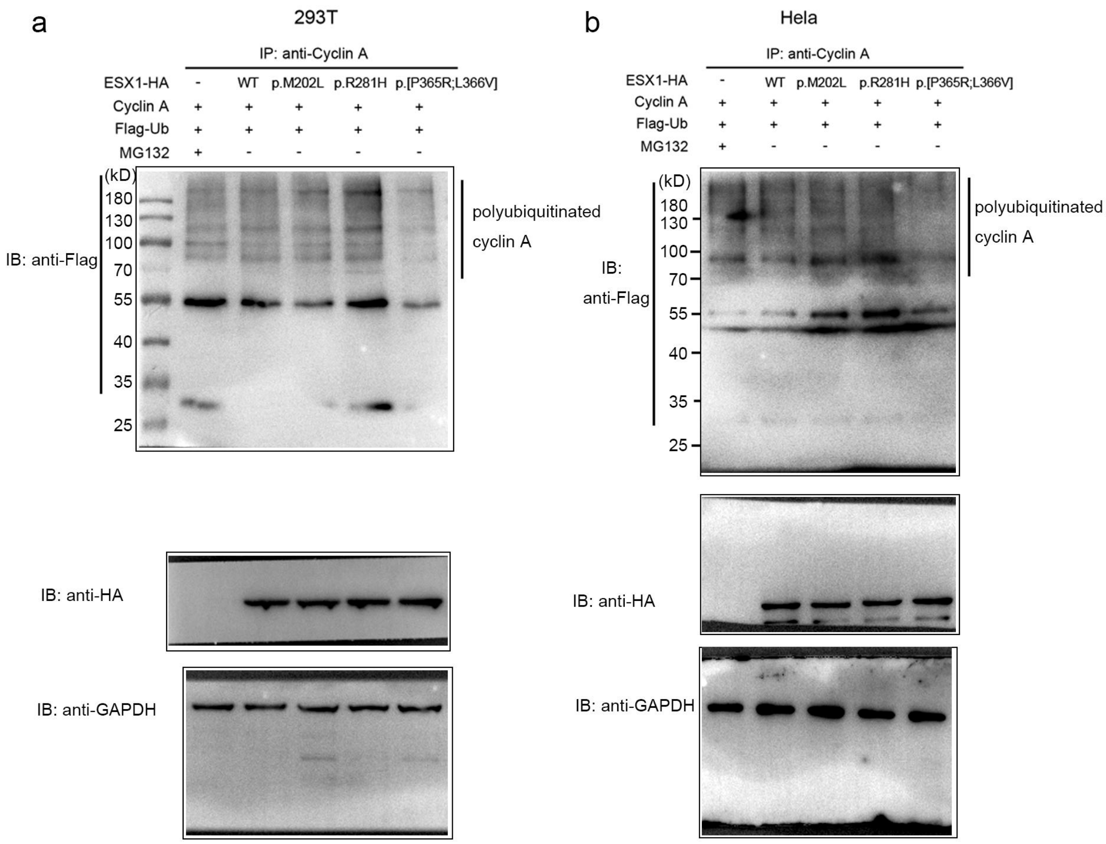

Figure S2. Repeated experiments of the effect of ESX1 mutations on cyclin A polyubiquitination in Hela cells (a). The intensity of poly-Ub bands and GAPDH bands was semi-quantified through Image J software, the ratios between them (poly-Ub/GAPDH) were analyzed statistically via GraphPad Prism 5. The results showed that compared with cells overexpressing WT ESX1, the relative poly-Ub levels in cells overexpressing p.[P365R; L366V] ESX1 was significantly decreased.

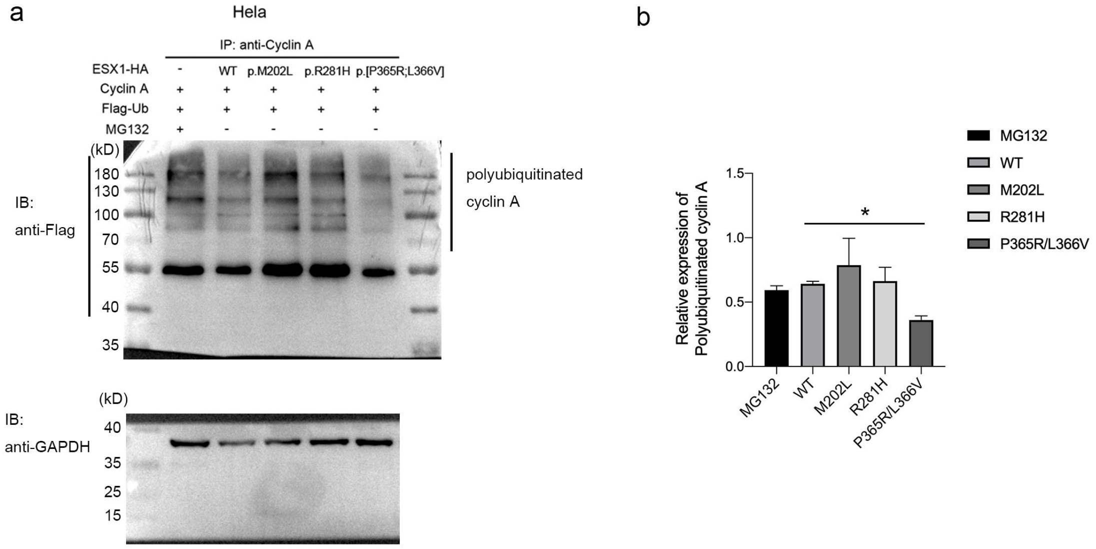

Figure S3. Full-length images of Western blots detecting the effect of p.P365R or p.L366V ESX1 on cyclin A polyubiquitination (Figure 4 a and b).

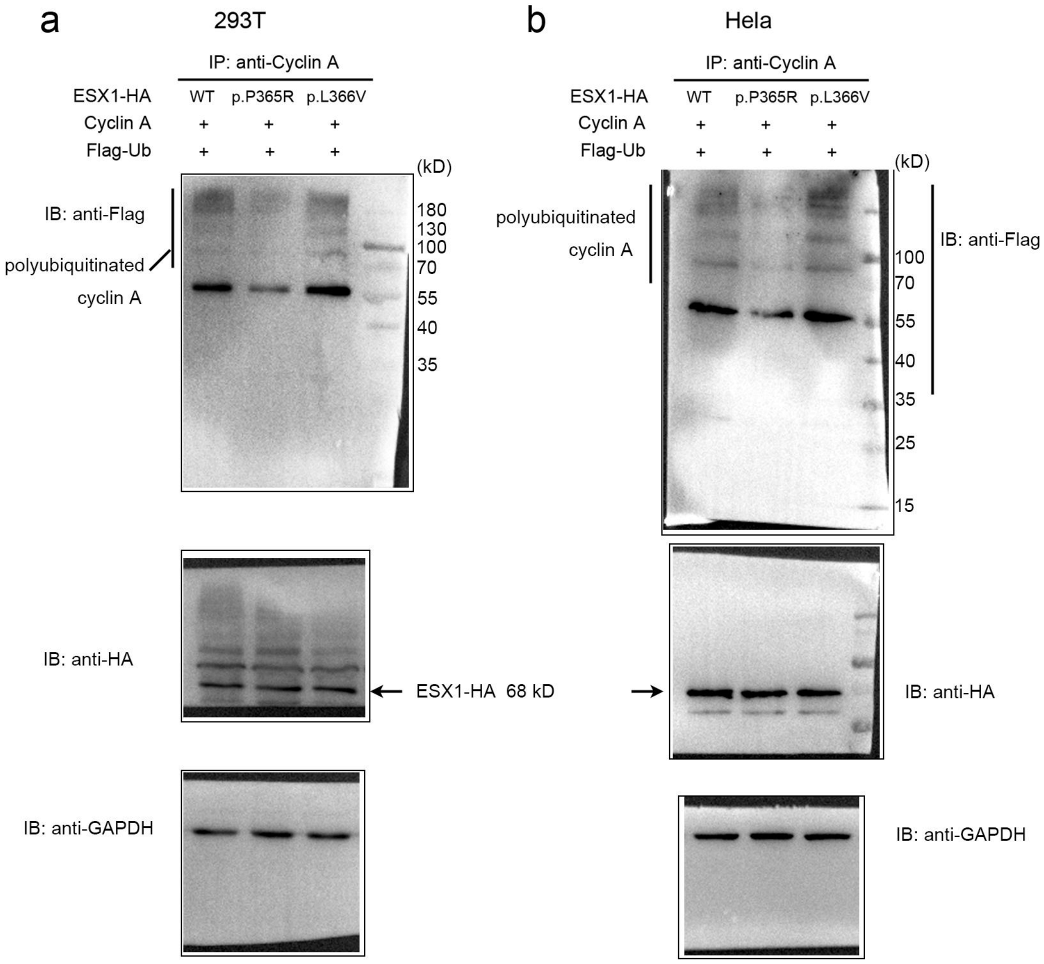

Figure S4. Testicular biopsy analysis of NOA patients harboring ESX1 p.M202L (W568) or p.R281H (W245) mutation.

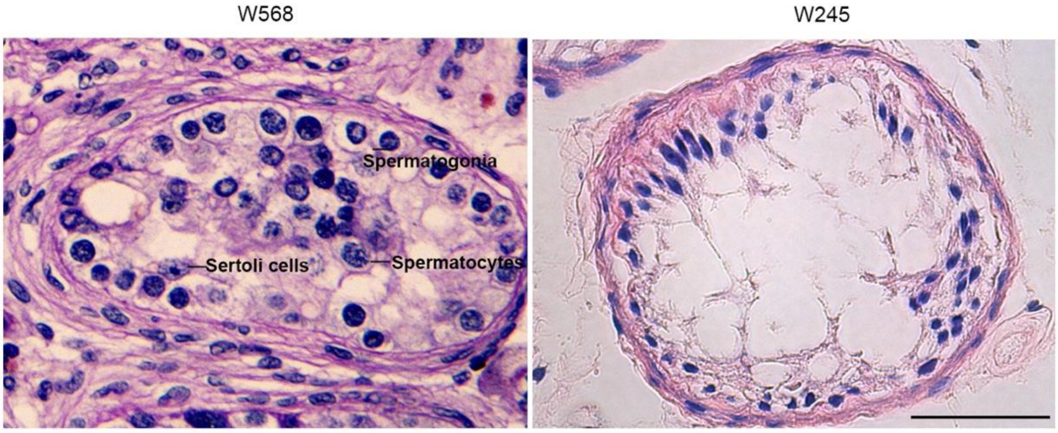

Fig. S4 Abnormal spermatogenesis was detected in NOA patients carrying ESX1 p.M202L (W568) or p.R281H (W245) mutation. Morphology of testes revealed that the spermatogenesis of patient W568 was arrested at the level of spermatocytes, while only Sertoli cells were observed in patient W245. Bar: 50 μm.
